# Association of Strength and Physical Functions in People with Parkinson's Disease

**DOI:** 10.1155/2018/8507018

**Published:** 2018-12-12

**Authors:** Sacha Clael, Elaine Brandão, Liana Caland, Raquel Techmeier, Tamara de Paiva, Jhonatan Rodrigues, Camila Wells, Lídia Bezerra

**Affiliations:** Faculty of Physical Education, University of Brasilia, Brasilia, Brazil

## Abstract

**Background:**

Parkinson's disease is responsible for decrease of activities of daily living and mobility limitations. Association of strength with physical capacities and disease time can improve training methodologies and predict changes in physical fitness for this population, since the control center of movements and strength is the same.

**Objective:**

Therefore, the aim of this study is to analyze if there are correlation between strength with functional tests (the sit-to-stand, the six-minute walk, and the timed-up-go) and disease time in people with Parkinson's disease.

**Results:**

All functional tests correlations are significant,* p* < 0.05. The strength is positively correlated with the sit-to-stand and the six-minute walk. The strength is negatively correlated with the timed-up-go.

**Conclusion:**

There are a correlation between strength with functional tests in people with PD, and changes in strength assessment can be used as predictor to changes in aerobic capacity.

## 1. Introduction

Parkinson's disease (PD) is a neurodegenerative disease characterized by progressive deterioration of the substantia nigra in the midbrain which cause a decrease in dopamine production [1], and this dopamine reduction may modify the somatic motor activities [2] in addition to diminish strength [3]. Moreover, people with PD can have tremor, rigidity, hypokinesia and postural instability that cause mobility loss and dependence to perform activities of daily life [4].

Quantify the motor symptoms of PD may aid on disease treatment, because once the disease progression was identify, professionals can be more accuracy to indicate appropriate treatments [5], and to identify some change, functional tests are used [6]. The 30 seconds sit-to-stand (STS30), the six-minute walk (6MW) and the timed-up-go (TUG) are often recommend for functional assessment [7], and also for people with PD [5, 8].

The 6MW is a practical way to assess aerobic capacity, the TUG evaluate agility and dynamic balance, and the STS30 determine lower body strength [7]. These three tests indicate the physical capacities necessary to perform activities of daily life [7]; furthermore, they are easy to learn and apply, have low cost, and are safe. Besides that, physical capacities may also be evaluated with equipment; for example, strength can be assessed by the peak torque (PT) on the isokinetic dynamometer [9].

The disease progression can be quantify also as severity or disease time, the literature shows association between disease severity with strength [10] or functional tests [11], but for the disease time the results are inconsistent [12].

For production of movements a muscular contraction mediated by the somatic nervous system, as well as strength, is necessary [2]. If the control center of movements and strength is the same, assess strength on the isokinetic dynamometer can be a predictor of changes in functional capacities on this population. Thus, the aim of the study is to check if there are correlation between strength with functional tests and disease time in people with PD. The hypothesis of the current study is that there will be a positive correlation between strength with the STS30 and the 6MW and a negative correlation between strength and the TUG.

## 2. Materials and Methods

### 2.1. Participants

A total of 34 individuals with PD were recruited and classified in one of four stages of the modified Hoehn and Yahr scale [13]. The subjects needed to visit laboratory 2 times, with 48 hours of interval, first day for anthropometric measurements and strength test, and second day for functional tests. Moreover, all the participants were evaluated in “*on*” medication period and instructed not to perform physical exercises on 24 hours prior the tests. This study was approved by the Faculty of Health Sciences at University of Brasilia ethics committee and all volunteers signed the consent form.

### 2.2. Anthropometric Measurements

To assess body composition, total fat mass, percentage of total body fat, and total fat free mass the DXA scan of the Prodigy (Lunar Corporation TM, Madison, WI, USA) was used, participants were on supine position and wearing light indoor clothing.

### 2.3. Isokinetic Dynamometer

To assess strength the Biodex system III Isokinetic Dynamometer (Biodex Medical, Inc., Shirley, NY) used with the protocol adapted from Bottaro et al [14]. To warm up and familiarization the voluntary performed one set of ten repetitions of knee extension and flexion at 120°/s with 60 seconds of rest interval. Then, to measure the PT and the relative peak torque (PT/BW) three sets of five repetitions of knee extension at 60°/s with 60 seconds of rest interval between sets were performed. The protocol was counterbalanced and performed in both legs.

### 2.4. Functional Tests

The Rikli & Jones protocols [7] were used to assess the STS30, the TUG, and the 6MW.

### 2.5. Statistical Analyses

For sample characterization, descriptive statistics were performed with mean and standard deviation for quantitative variables and simple frequency for qualitative variables. To verify data normality the Shapiro-Wilk test was used. To correlate the strength with the STS30 and diagnostic time Pearson's test was performed, and to correlate the strength with the TUG and the 6MW the Spearman's test was used. Statistical significance level was set at* p* ≤ 0.05 in all correlations. All analyses were performed using the SPSS 24 (IBM Corporation, Armonk, NY, USA, 24.0) for Windows.

## 3. Results

Sample characterizations are described in detail in Table 1.

Table 2 shows the correlations between strength with functional tests and diagnostic time. For functional tests, all correlations are significant,* p* < 0.05. The strongest correlation is between the 6MW and the PT/BW on right side at second set (*rho* = 0.617), and the lowest correlation is between the TUG and the PT/BW on right side at second set (*rho* = -0.363). For diagnostic time, the strongest correlation is on PT on left side at first set (*r* = 0.353), and the lowest correlation is on PT/BW on right side at third set (*r* = 0.167).

Tables 3 and 4 show the correlation between strength with functional tests and diagnostic time for men and women, respectively. On Table 3 for functional tests, the strongest correlation is between the 6MW and the PT/BW on right side at second set (*rho* = 0.617), and the lowest correlation is between the TUG and the PT/BW on left side at second set (*rho* = -0.499). For diagnostic time, the strongest correlation is on PT on left side at first set (*r* = 0.449), and the lowest correlation is on PT/BW on right side at third set (*r* = 0.199).

On Table 4 for functional tests, the strongest correlation is between the STS30 and the PT/BW on left side at second set (*r* = 0.945), and the lowest correlation is between the TUG and the PT on left side at second set (*rho* = -0.898). For diagnostic time, the strongest correlation is on PT on left side at second set (*r* = 0.203), and the lowest correlation is on PT/BW on right side at first set (*r* = -0.018).

## 4. Discussion

The results show that there are correlation between strength and functional tests in people with PD; therefore this association shows that physical capacities are dependent on this population. The main finding is that all the strength are not correlated with diagnostic time, and for functional tests the results associations are moderate and strong, showing the good relation between the variables. Thus, determine values of strength and functional tests can help to identify individuals with an higher risk of impairment and also collaborate on prescription of physical exercises focusing on capacities that each individual is losing [15].

The decrease in muscular strength as a symptom of PD can begin in adolescence [16], and identify such symptom can anticipate the clinical treatment, but only 4 variables are correlated with the diagnostic time, and when the group was split by gender, just men have some significant results, which confirms that the duration of PD is not associated with strength [12]. However, physical activity level of the sample was unknown, which was known is that they practiced some physical activity, but not the intensity neither how long, so if they did exercise for a long time, this can be a protective factor [17]. Moreover, in the aging process men lose almost twice as much strength as women [18], which perhaps explains only men having some significant results in diagnostic time.

Regarding to the results variations between men and women, the sample stratification can cause a type 2 error, due to sample reduction within the groups [19], decreasing the statistical power. Moreover, the change of strongest correlation of 6MW to STS30 perhaps is explained because men have difficulty to initiate movements [20], and STS30 is a time test.

The isokinetic dynamometer is considered a gold standard on strength assessment [21], and the PT correlated with the functional variables shows that changes in strength may directly reflect on the aerobic capacity, agility, and dynamic balance. Moreover, variations in strength or functional tests can show how fast the disease progression is, and people with PD have reduced mobility because of their muscular strength, which may increase the fall risk, since the smaller the force produce, the lower the functionality of this population [22]

Positive association between strength and STS30 was expected; because both tests are used to assess strength, this result shows that the greater the subject strength the more times will sit on the bench. The equipment is more sophisticated [23], but the functional test is more easy and cheap to applicable [24]; therefore assessing the individual clinical progress of strength the STS30 is a good choice.

Negative correlation of the strength with the TUG show that the greater the subject strength, the shorter the time to perform the test, moreover individuals with high TUG scores have greater chance of fall and imbalance, which can carry on fear to perform physical exercises, getting worse quality of life and functionality [25]. Correlation between the TUG and the strength shows that if strength is maintained, agility and dynamic balance are preserved, because the TUG require volunteer's agility [7], so the better the score the lower the bradykinesia [26] and the hypokinesia [27].

A surprising finding is the association between a strength test and an aerobic capacity test; appropriate results on the 6MW are an indicator that individual has an exercise habit and a good strength despite the disease stage [8], which shows an improvement of respiratory capacity and distance traveled. However, people with PD in moderate to severe stages can have a decrease in maximal oxygen consumption, but this is still in debate in the literature [28].

Blend two or more physical capacities in a training program, it tends to have a better result [29]. To practical application, based on the dependent relationships between variables, a training program that involves strength, balance, and aerobic capacity should be used for clinical treatment.

There are a few limitations associated with the present study. The first limitation is the small sample size which reduces the statistical power and the generalizability of our findings. The second is not split individuals by stages of the modified Hoehn and Yahr scale neither by level of physical activity. Future studies could analyze each stages of the modified Hoehn and Yahr scale separately, increase the sample size, add more functional tests, and assess this population in the “*off*” medication period.

## 5. Conclusion

There is a correlation between strengths with functional tests in people with PD and changes in strength assessment can be used as predictor to changes in aerobic capacity.

## Figures and Tables

**Table 1:** Sample characterization (*n* = 34).

	**Mean ± SD**

Age (years)	67.36 ± 8.87
Body Mass (kilograms)	73.83 ± 14.75
Height (meters)	1.67 ± 0.08
Total Fat Mass	23.17 ± 9.32
Body Fat (%)	31.89 ± 9.25
Total Fat Free Mass	47.50 *± *7.79
Diagnostic time (years)	11 *±* 4.95
**Gender (*f)***	***f (***%**)**
Men	26 (76.5)
Women	8 (23.5)
**Modified Hoehn & Yard *(f)***	***f (***%**)**
Level – 1.0	4 (11.8)
Level – 1.5	2 (5.9)
Level – 2.0	19 (55.9)
Level – 2.5	3 (8.8)
Level – 3.0	5 (14.7)
Level – 4.0	1 (2.9)

SD = standard deviation; *f* = frequency; % = percentage.

**Table 2:** Correlation of PT and PT/BW with functional tests and diagnostic time.

	Diagnostic Time		STS30		TUG		6MW	
	*r*	*p*	*r*	*p*	*rho*	*p*	*rho*	*p*

PT R $1^{\underline{a}}$	0.327	0.059	0.478	0.004	- 0.460	0.005	0.572	≤ 0.001
PT/BW R $1^{\underline{a}}$	0.194	0.272	0.483	0.003	- 0.416	0.013	0.591	≤ 0.001
PT R $2^{\underline{a}}$	0.319	0.066	0.404	0.016	- 0.409	0.015	0.587	≤ 0.001
PT/BW R $2^{\underline{a}}$	0.199	0.258	0.407	0.015	- 0.363	0.032	0.617^*∗*^	≤ 0.001
PT R $3^{\underline{a}}$	0.297	0.088	0.437	0.009	- 0.466	0.005	0.587	≤ 0.001
PT/BW R $3^{\underline{a}}$	0.167	0.345	0.435	0.009	- 0.374	0.027	0.569	≤ 0.001
PT L $1^{\underline{a}}$	0.353	0.041	0.428	0.010	- 0.540	0.001	0.447	0.008
PT/BW L $1^{\underline{a}}$	0.247	0.159	0.447	0.007	- 0.523	0.001	0.495	0.003
PT L $2^{\underline{a}}$	0.349	0.043	0.437	0.009	- 0.568	≤ 0.001	0.462	0.006
PT/BW L $2^{\underline{a}}$	0.229	0.192	0.448	0.007	- 0.543	≤ 0.001	0.499	0.003
PT L $3^{\underline{a}}$	0.335	0.053	0.445	0.007	- 0.533	≤ 0.001	0.443	0.009
PT/BW L $3^{\underline{a}}$	0.207	0.241	0.449	0.007	- 0.500	0.002	0.459	0.006

STS30 = 30 seconds sit-to-stand; TUG = timed-up-go; 6MW = six-minute walk; PT = peak torque absolute; PT/BW = peak torque relative; R = right side; L = left side; $1^{\underline{a}}$, $2^{\underline{a}}$, and $3^{\underline{a}}$ = order sets; *∗* = strongest correlation.

**Table 3:** Correlation of PT and PT/BW with functional tests and diagnostic time in men.

	Diagnostic Time		STS30		TUG		6MW	
	*r*	*p*	*r*	*p*	*rho*	*p*	*rho*	*p*

PT R $1^{\underline{a}}$	0.428	0.029	0.441	0.024	-0.445	0.023	0.566	0.003
PT/BW R $1^{\underline{a}}$	0.256	0.207	0.406	0.040	-0.358	0.073	0.548	0.005
PT R $2^{\underline{a}}$	0.397	0.045	0.373	0.061	-0.379	0.056	0.595	0.002
PT/BW R $2^{\underline{a}}$	0.248	0.223	0.351	0.079	-0.337	0.092	0.617^#^	≤ 0.001
PT R $3^{\underline{a}}$	0.370	0.062	0.407	0.039	-0.435	0.026	0.579	0.002
PT/BW R $3^{\underline{a}}$	0.199	0.329	0.370	0.063	-0.341	0.088	0.561	0.004
PT L $1^{\underline{a}}$	0.449	0.022	0.373	0.060	-0.491	0.011	0.336	0.100
PT/BW L $1^{\underline{a}}$	0.302	0.134	0.355	0.075	-0.470	0.015	0.365	0.072
PT L $2^{\underline{a}}$	0.438	0.025	0.371	0.062	-0.499	0.009	0.364	0.074
PT/BW L $2^{\underline{a}}$	0.270	0.182	0.344	0.085	-0.458	0.018	0.347	0.089
PT L $3^{\underline{a}}$	0.441	0.024	0.386	0.052	-0.478	0.013	0.342	0.094
PT/BW L $3^{\underline{a}}$	0.267	0.188	0.350	0.080	-0.424	0.031	0.329	0.108

STS30 = 30 seconds sit-to-stand; TUG = timed-up-go; 6MW = six-minute walk; PT = peak torque absolute; PT/BW = peak torque relative; R = Right side; L = left side; $1^{\underline{a}}$, $2^{\underline{a}}$, and $3^{\underline{a}}$ = order sets; # = strongest correlation.

**Table 4:** Correlation of PT and PT/BW with functional tests and diagnostic time in women.

	Diagnostic Time		STS30		TUG		6MW	
	*r*	*p*	*r*	*p*	*rho*	*p*	*rho*	*p*

PT R $1^{\underline{a}}$	0.085	0.840	0.920	≤ 0.001	-0.548	0.160	0.619	0.102
PT/BW R $1^{\underline{a}}$	- 0.018	0.966	0.923	≤ 0.001	-0.619	0.102	0.714	0.47
PT R $2^{\underline{a}}$	0.150	0.723	0.720	0.044	-0.619	0.102	0.667	0.071
PT/BW R $2^{\underline{a}}$	0.034	0.936	0.723	0.043	-0.595	0.120	0.667	0.071
PT R $3^{\underline{a}}$	0.198	0.638	0.776	0.024	-0.714	0.047	0.738	0.037
PT/BW R $3^{\underline{a}}$	0.100	0.815	0.801	0.017	-0.595	0.120	0.667	0.071
PT L $1^{\underline{a}}$	0.155	0.714	0.911	0.002	-0.778	0.023	0.850	0.007
PT/BW L $1^{\underline{a}}$	0.087	0.838	0.914	0.002	-0.778	0.023	0.850	0.007
PT L $2^{\underline{a}}$	0.203	0.629	0.924	≤ 0.001	-0.898	0.002	0.922	≤ 0.001
PT/BW L $2^{\underline{a}}$	0.130	0.758	0.945^$^	≤ 0.001	-0.826	0.011	0.898	0.002
PT L $3^{\underline{a}}$	0.076	0.858	0.908	0.002	-0.707	0.050	0.731	0.040
PT/BW L $3^{\underline{a}}$	- 0.007	0.987	0.907	0.002	-0.707	0.050	0.731	0.040

STS30 = 30 seconds sit-to-stand; TUG = timed-up-go; 6MW = six-minute walk; PT = peak torque absolute; PT/BW = peak torque relative; R = right side; L = left side; $1^{\underline{a}}$, $2^{\underline{a}}$, and $3^{\underline{a}}$ = order sets; $ = strongest correlation.

## Data Availability

The data used to support the findings of this study are available from the corresponding author upon request.
